# Effect of Community Affluence on the Association Between Individual Socioeconomic Status and Cardiovascular Disease Risk Factors, Colorado, 2007–2008

**DOI:** 10.5888/pcd9.110305

**Published:** 2012-06-21

**Authors:** Ian Matthew Abeyta, Nicole R. Tuitt, Tim E. Byers, Angela Sauaia

**Affiliations:** Author Affiliations: Ian Matthew Abeyta, Nicole R. Tuitt, Colorado School of Public Health, UCD, Denver, Colorado; Tim E. Byers, Colorado School of Public Health, UCD, University of Colorado Cancer Center, Denver, Colorado.

## Abstract

We assessed the hypothesis that community affluence modifies the association between individual socioeconomic status (SES) and 6 cardiovascular disease (CVD) risk factors: diabetes, hypertension, physical inactivity, obesity, smoking, and poor nutrition. We stratified data from the Colorado Behavioral Risk Factor Surveillance System for 2007 and 2008 by individual SES and 3 categories of community affluence (median household income of county). People who had a low SES seemed to benefit from residing in high-affluence communities. Living in high-affluence communities may mitigate the effect of poverty on CVD risk factors; our findings support the value of interventions that address social determinants of health.

## Objective

Evidence demonstrates that socioeconomic status (SES) is associated with cardiovascular disease (CVD) in large part through modifiable risk factors such as obesity, tobacco use, and sedentary lifestyle (1,2). Several recent studies have explored the association between neighborhood deprivation and risk factors and chronic disease incidence and mortality (3-6). Whereas prevention efforts that focus on individual characteristics that control behavior are important, environmental and social elements also affect personal choices, are modifiable risk factors (7,8), and deserve attention. The objective of this study was to assess how community affluence, as measured by median household income, modifies the association between individual SES and 6 CVD risk factors.

## Methods

We analyzed Colorado Behavioral Risk Factor Surveillance System (BRFSS) data for 2007 and 2008 stratified by 3 categories of individual household income (predefined by BRFSS): low SES (<$25,000); medium SES ($25,000–$49,999), and high SES (≥$50,000). In 2007 and 2008, according to BRFSS data on 20,739 Colorado residents, 19% were low SES, 24% were medium SES, and 57% were high SES. We examined the prevalence of the following 6 CVD risk factors: diabetes (“ever been told that had diabetes,” excluding gestational diabetes); hypertension (“ever been told that had high blood pressure,” excluding gestational hypertension); physical inactivity (“participated in any physical activities other than regular job during past 30 days”); obesity (body mass index [BMI] ≥30); smoking (“current cigarette smoking”); and average fruit and vegetable consumption per day (≥5 servings/d or <5 servings/d [data available for 2007 only]). BMI (in kg/m^2^) was calculated from self-reported height and weight. 

As a measure of community affluence, we used county median household income from the most recent 5-year (2005–2009) American Community Survey estimates. We classified median income into 3 groups: low ($23,041–$39,992 [21 counties]); medium ($40,658–$54,909 [22 counties]); and high ($55,258–$99,522 [21 counties]). We used these tertiles to create a comparable number of counties in each range.

We examined the influence of community affluence on the association between individual SES and CVD risk factors through multiple regression models by adding an interaction term and adjusting for median age of community. We expressed data as median and interquartile range and used SAS version 9.3 (SAS Institute, Inc, Cary, North Carolina) for all analyses. Significance was set at *P* < .05.

## Results

The prevalence of CVD risk factors in the low-SES group was lower among people living in high-affluence communities than in people living in low-affluence communities (Table). In the low-SES group, after adjustment for age, community affluence was significantly associated with diabetes (*P* = .01) and consumption of less than 5 servings of fruits and vegetables per day (*P* = .04). In the medium-SES group, none of the age-adjusted associations between community affluence and risk factors were significant. In the high-SES group, we found an age-adjusted association between community affluence and no exercise (*P* = .04).

**Table:** Prevalence of Risk Factors for Cardiovascular Disease, by Individual Socioeconomic Status (SES)^a^ and Community Affluence^b^, and Multiple Regression Parameter Estimate for Community Affluence^c^

Risk Factor by Individual SES	High Affluence, Median % (IQR)	Medium Affluence, Median % (IQR)	Low Affluence, Median % (IQR)	β2	*P* Value
**Diabetes**					
Low	6.9 (5.0–11.4)	6.1 (4.0–8.8)	12.2 (7.9–17.8)	−1.31	.01
Medium	6.2 (4.4–7.0)	4.6 (2.6–6.9)	5.8 (2.7–8.1)	0.24	.78
High	2.4 (2.1–3.8)	3.5 (3.0–4.6)	3.2 (0–7.3)	−1.00	.25
**Hypertension**					
Low	23.1 (20.5–34.0)	25.2 (22.4–36.7)	29.7 (22.8–49.0)	−2.36	.55
Medium	21.4 (16.6–23.1)	18.1 (14.1–26.8)	35.7 (27.0–36.1)	−3.30	.34
High	18.5 (14.9–19.0)	18.8 (16.4–21.8)	16.7 (15.2–28.8)	0.43	.82
**No exercise**					
Low	29.6 (23.9–38.5)	28.9 (16.1–32.5)	36.0 (32.0–40.1)	−1.42	.58
Medium	19.6 (14.2–24.0)	22.4 (19.6–28.1)	18.6 (13.0–29.7)	−2.20	.22
High	11.0 (7.7–12.5)	11.0 (7.7–12.4)	12.7 (7.0–18.0)	−3.37	.04
**Obesity**					
Low	21.6 (17.6–25.3)	24.1 (16.7–25.4)	28.0 (17.7–37.2)	−3.00	.15
Medium	19.2 (14.0–26.7)	18.2 (15.6–21.3)	30.5 (2.0–32.0)	−3.91	.06
High	16.6 (16.6–20.7)	14.4 (10.0–22.7)	25.2 (22.9–34.7)	−5.58	.01
**Smoking**					
Low	22.6 (11.3–27.5)	29.2 (25.0–41.1)	30.9 (25.6–38.6)	−1.57	.09
Medium	25.2 (16.2–31.3)	18.8 (18.5–25.4)	26.1 (16.7–27.2)	1.24	.60
High	11.6 (9.0–13.1)	13.9 (9.9–16.9)	12.2 (6.5–18.1)	−0.66	.59
**<5 Servings of fruits and vegetables per day**					
Low	77.3 (74.8–85.5)	81.6 (77.5–82.3)	91.8 (82.3–97.2)	−6.17	.04
Medium	76.0 (72.6–77.2)	70.9 (68.7–77.6)	84.5 (77.1–95.2)	−1.10	.77
High	73.1 (65.7–74.9)	72.2 (70.5–75.3)	83.8 (53.2–86.4)	−3.34	.26

Abbreviations: IQR, interquartile range.

^a^ Individual SES categorized as low, annual household income of <$25,000; medium, $25,000–$49,999, and high, ≥$50,000.

^b^ Community affluence categorized according to county median household income as low, $23,041–$39,992 (21 counties); medium, $40,658–$54,909 (22 counties); and high, $55,258–$99,522 (21 counties).

^c^ Predicted Risk Factor Percentage = Intercept + β1 * County Median Age + β2 * Community Affluence, where Community Affluence is coded as 0 for low, 1 for medium, and 2 for high. Parameter estimate adjusted for median age in county.

**Figure:**
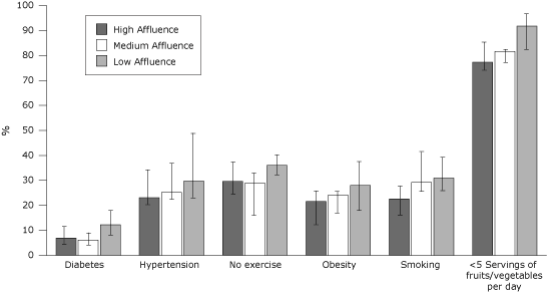
Prevalence of cardiovascular disease risk factors among people identified as having low socioeconomic status (annual household income of <$25,000), by community affluence. Community affluence was categorized according to county median household income: low ($23,041–$39,992 [21 counties]); medium ($40,658–$54,909 [22 counties]); and high ($55,258–$99,522 [21 counties]). Error bars represent interquartile ranges.

**Table:** 

Risk Factor	High Level of Affluence, Median (Interquartile Range)	Medium Level of Affluence, Median (Interquartile Range)	Low Level of Affluence, Median (Interquartile Range)
**Diabetes**	6.9 (5.0–11.4)	6.1 (4.0–8.8)	12.2 (7.9–17.8)
**Hypertension**	23.1 (20.5–34)	25.2 (22.4–36.7)	29.7 (22.8–49)
**No exercise**	29.6 (23.9–38.5)	28.9 (16.1–32.5)	36 (32.0–40.1)
**Obesity**	21.6 (17.6–25.3)	24.1 (16.7–25.4)	28 (17.7–37.2)
**Smoking**	22.6 (11.3–27.5)	29.2 (25.0–41.1)	30.9 (25.6–38.6)
**<5 Servings of fruits/vegetables per day**	77.3 (74.8–85.5)	81.6 (77.5–82.3)	91.8 (82.3–97.2)

## Discussion

Our analysis of CVD risk factors suggests that people who have low SES may benefit from living in high-affluence communities. Although these behaviors are often approached as personal choices, our findings as well as others’ underscore the potential influence of external factors on the individual risk profile (3,5,6,9-12). Resources in high-affluence communities, particularly access to healthy food, health care services, and physical activity opportunities, may allow economically disadvantaged residents to adopt a healthy lifestyle.

Adjustment for differences in age distribution affected some of the associations between community affluence, individual SES, and risk factor prevalence. Our BRFSS data showed that low-, medium-, and high-SES groups had similar proportions of people aged 45 years or younger (53%, 51%, 50%, respectively), whereas data from the ACS 2005–2009 showed that residents in low-affluence communities were older than their counterparts in medium- and high-affluence communities (median age 41 y, 39 y, and 37 y, respectively).

Our study has limitations. We examined data from only 1 state and from only 2 years, and the analysis was ecological. In addition, we did not account for other community affluence–related factors, such as access to healthy food, health care services, and physical activity opportunities. Nevertheless, our findings suggest that the interaction between community affluence and individual SES should be further investigated in larger samples and by adjusting for other characteristics.

Community affluence may mitigate the effect of low income on several modifiable CVD risk factors. Data for such analyses are easily available online and can be used to further explain the associations between chronic disease risk factors and social determinants of health. These findings have health policy and data monitoring implications.
